# Measurement of Rapid Amiloride-Dependent pH Changes at the Cell Surface Using a Proton-Sensitive Field-Effect Transistor

**DOI:** 10.3390/bios6020011

**Published:** 2016-03-30

**Authors:** Daniel Schaffhauser, Michael Fine, Miyuki Tabata, Tatsuro Goda, Yuji Miyahara

**Affiliations:** 1 Institute of Biomaterials & Bioengineering, Tokyo Medical and Dental University, Bldg 21-4-403B, 2-3-10 Kanda-Surugadai, Chiyoda, Tokyo 101-0062, Japan; danielschaffhauser@gmail.com (D.S.); tabata.bsr@tmd.ac.jp (M.T.); goda.bsr@tmd.ac.jp (T.G.); 2 Department of Physiology, UT Southwestern Medical Center, 5323 Harry Hines Blvd., Dallas, TX 75390, USA; michael.fine@utsouthwestern.edu

**Keywords:** ISFET, pH, proton, CHO, MSF, ammonia, amiloride, EIPA, NHE

## Abstract

We present a novel method for the rapid measurement of pH fluxes at close proximity to the surface of the plasma membrane in mammalian cells using an ion-sensitive field-effect transistor (ISFET). In conjuction with an efficient continuous superfusion system, the ISFET sensor was capable of recording rapid changes in pH at the cells’ surface induced by intervals of ammonia loading and unloading, even when using highly buffered solutions. Furthermore, the system was able to isolate physiologically relevant signals by not only detecting the transients caused by ammonia loading and unloading, but display steady-state signals as would be expected by a proton transport-mediated influence on the extracellular proton-gradient. Proof of concept was demonstrated through the use of 5-(*N*-ethyl-*N*-isopropyl)amiloride (EIPA), a small molecule inhibitor of sodium/hydrogen exchangers (NHE). As the primary transporter responsible for proton balance during cellular regulation of pH, non-electrogenic NHE transport is notoriously difficult to detect with traditional methods. Using the NHE positive cell lines, Chinese hamster ovary (CHO) cells and NHE3-reconstituted mouse skin fibroblasts (MSF), the sensor exhibited a significant response to EIPA inhibition, whereas NHE-deficient MSF cells were unaffected by application of the inhibitor.

## 1. Introduction

**Figure 1:**
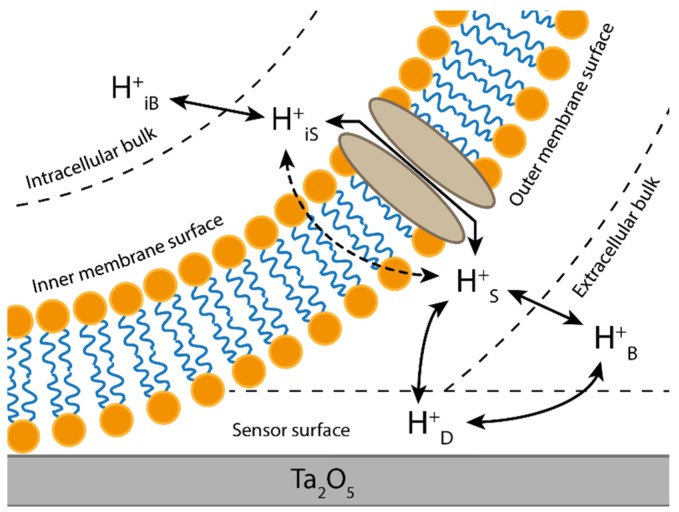
Simplified model of the proton pathways near the plasma membrane and the sensor surface. Proton translocation across the membrane is possible either through an active transporter protein or gated channel (**solid arrow**) or via direct diffusion across the lipid-bilayer (**dotted arrow**). In reality, charged ions and protons are unlikely to diffuse across the lipid-bilayer. However, some molecules can freely diffuse across the bilayer in their uncharged state. If these molecules associate with free protons, they can leave behind or sequester protons as they diffuse back and forth across the membrane changing the net proton balance without actual proton translocation.

Proton-dependent membrane transport plays a significant role in many physiological processes, such as pH regulation in blood and tissue, maintenance of cellular volume, glycolysis or as a co-requisite for the transport and absorption of amino acids, peptides and iron [1,2]. These processes generate changes in extracellular proton gradients that are difficult to detect as the length of proton flux is largely determined by the buffering capacity of the extracellular bulk solution [3]. Improvement of the pH signal strength can thus be achieved through reduction of the buffering strength of the extracellular bulk solution or by measuring the pH in close proximity to the cell membrane. However, apart from the proton’s ability to diffuse through water much faster than other ions, it has been discovered that protons are able to migrate still faster along the outside of lipid bilayers with relative ease. Several experiments have demonstrated how protons travel across large portions of a membrane before diffusing into the bulk solution. One study on the purple membrane of *H. salinarium* involved the use of Pyranine as a fluorescent pH indicator where the time constant for transfer of a proton along the membrane surface was shown to be almost six times faster than the one to the bulk solution [4]. In another study, double patch clamping was used to measure proton flux between two integral gramicidin channels, where it was found that protons could migrate along the membrane up to a distance of 200 µm [5]. Subsequently, a mathematical model for proton diffusion along biological membranes was developed by Medvedev and Stuchebrukhov which also included the influence of the buffering strength of the bulk solution on the surface proton retardation [6]. Using these principals of proton behavior along the surface of cell, we demonstrated in previous experiments that it is possible to monitor pH-dependent transport through membrane proteins heterologously expressed in large *Xenopus laevis* oocytes using an ion-sensitive field-effect transistor (ISFET) designed for proton detection [7]. Furthermore, we demonstrated that we could achieve so even when using strongly buffered solutions under constant superfusion. We took advantage of the large surface area of an oocyte and isolated a section of the membrane tightly against the sensor’s surface, restricting the interface from the buffered superfusion. Under these conditions, pH-sensing was still possible, suggesting significant proton coupling between the exposed and isolated membrane sections as one would expect if protons freely “hopped” along the surface of the cell before diffusing into bulk solution. For this method, the ISFET utilized a high proton affinity Ta_2_O_5_ gate insulator generating significant specificity for proton detection with little interference from other ions [8]. As modeled in Figure 1, we identify the possible pathways of protons near and inside the cell. These pathways are essentially created via proton sources and sinks, all characterized by their ability to buffer protons reasonably well. In the present study, these sources and sinks are created by the inner and outer surfaces of the plasma membrane, the bulk solution and the sensor itself.

Based on our previous experimental findings and success with the ISFET sensor, we developed a system for the measurement of pH at the surface of immobilized mammalian cells. It has previously been demonstrated on tumor cells that it is possible to measure their extracellular proton gradient during transient perfusion using an Al_2_O_3_ ISFET and after permeabilization with TX100 [9]. However, only relatively slow changes in pH could be measured. In our study, we monitored rapid pH fluxes of continuously superfused cells utilizing ammonia to briefly acidify the intracellular environment and stimulate the release of protons into the extracellular space through the sodium-coupled transport of sodium/hydrogen exchangers (NHEs). The high membrane permeability of uncharged ammonia allows the transmembranal proton balance to be altered temporarily by alternating intervals of ammonia loading and unloading [10]. During unloading, uncharged ammonia diffuses freely across the membrane into the extracellular space leaving behind a proton resulting in a net acidification of the intracellular environment. The resulting acidification of the cell is subsequently compensated through outward NHE transport of excess protons. The activation of NHEs is dependent on an inward transmembrane sodium gradient as the energy source and inhibited by the amiloride derivative 5-(*N*-ethyl-*N*-isopropyl)amiloride (EIPA) [11]. Amiloride and its derivatives are biophysical substitutes for sodium and thus inhibit NHE through competitive binding in the sodium binding pocket [12,13]. Being a diuretic, amiloride is therapeutically used to treat a number of medical conditions such as hypertension or congestive heart failure [14,15,16,17]. We opted for EIPA due to its increased affinity for NHE over other sodium-coupled transporters and high effectiveness in blocking NHE in the low-micromolar range, thus lowering the probability of interference with the sensor signal [18]. After careful optimization of the experimental parameters, it was possible to record strong pH-dependent signals near the cellular membrane, with the ability to resolve fast transients occurring during the ammonia loading and unloading intervals. EIPA-sensitive signals were indeed recorded in Chinese hamster ovary (CHO) cells and NHE3-reconstituted mouse skin fibroblasts (MSF), whereas NHE-deficient MSF were largely devoid of any EIPA response.

## 2. Materials and Methods

### 2.1. System Setup

**Figure 2:**
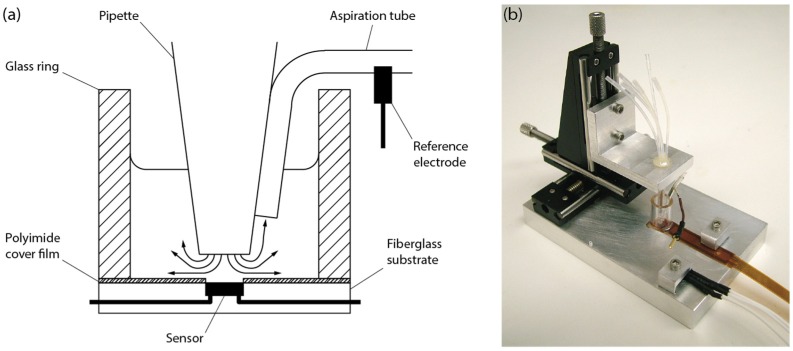
(**a**) Graphical representation of the integration concept; (**b**) Photographic image of the unconnected setup.

The sensor consisted of an *n*-channel field-effect transistor (FET) with Ta_2_O_5_ as a gate insulator of 40 nm thickness and no metalization (ISFETCOM Co. Ltd., Hidaka, Japan). The sensor was commercially available for industrial pH applications provided in a complete package consisting of a fiberglass substrate and a polyimide cover with a window to prevent wetting of the drain and source contacts. Integration of the sensor with the solutions handling system was achieved by adhering a glass ring to the sensor top, effectively forming a reservoir of approximately 0.1 mL. A perfusion pipette containing a bundle of silica capillary tubes was mounted onto a manual two-axis positioning stage, in order to facilitate alignment of the pipette with the sensor. The individual capillary tubes were connected to a system comprising a six-fold syringe pump and three-way electrically actuated valves for channel selection. A tube for aspiration was fixed to the pipette with its opening approximately 2 mm away from the opening of the pipette. An additional syringe pump was connected to the aspiration tube to create a sink for the superfusion system. A reference electrode consisting of a chlorinated silver wire was inserted into the aspiration tube, functioning as a means for grounding the bulk solution. A schematic representation of the complete assembly is shown in Figure 2a. The assembly was mounted onto an aluminum base plate comprising mounting holes for the positioning stage onto which the pipette was mounted and a precisely machined rectangular recession for reproducible alignment of the sensor with the pipette (Figure 2b). A temperature sensor with continuous readout capability was mounted onto the base plate to ensure that the experiment was conducted at the desired temperature of 37 °C, achieved by placing the entire apparatus into an enclosed incubator. For sensor readout, the FET was operated as a source-drain follower, as described earlier [19]. The drain to source voltage was set to 0.5 V and the drain current was set to 0.5 mA. A high-resolution data-acquisition system with 50/60 Hz rejection was connected for recording the voltage between drain and reference (LabJack U6-Pro, LabJack Corp., Lakewood, CO, USA). User control and automation of the experiment was achieved through our proprietary software written in Visual Basic NET.

### 2.2. Cell Culture

Chinese hamster ovary (CHO) cells were maintained in Dulbecco’s Modified Eagle Medium: Nutrient Mixture F-12 (DMEM/F12) (ThermoFisher Scientific, Waltham, MA, USA) supplemented with 10% fetal bovine serum (FBS) (Atlanta Biologicals, Norcross, GA, USA), non-essential amino acids, 4 mM l-Glutamine, 10 mg/L sodium pyruvate and 100 units per mL of the antibiotics penicillin and streptavidine (Sigma-Aldrich, St. Louis, MO, USA). Cells were cultured in a humidified 37 °C cell-culture incubator with 5% CO_2_. Mouse skin fibroblasts (MSF) were maintained and supplemented as described above using DMEM as the base medium. Passaging of cells was performed at approximately 80% confluence. For each passage and prior to experimentation, cells were washed in Dulbecco’s phosphate-buffered saline (ThermoFisher Scientific, Waltham, MA, USA) and trypsinized in a Ca^2+^- and Mg^2+^-free solution for 5 min using a trypsin concentration of 0.25% weight per volume (Gibco).

### 2.3. Solutions and Reagents

An isotonic solution similar to Ringer’s solution containing 140 mM NaCl, 4 mM KCl and 1 mM MgCl_2_, adjusted to a pH of 7.4, was used as a base solution for all experiments. Buffering of the solutions was achieved through the addition of either 1 mM or 10 mM bis-tris propane (BTP), a buffer known for its very wide buffering range. Sucrose was added to solutions to maintain equiosmolar conditions and prevent any influence of osmosis and cellular shrinking or swelling on the signal. For the solutions containing ammonia, 20 mM of NH_4_Cl was added and any pH shift was accounted for by titration with NaOH. In addition, 10 µM EIPA (Sigma-Aldrich) was added to the final buffered solution for NHE-inhibition.

### 2.4. Sample Preparation and Experimental Procedure

The sensor surface was cleaned using a solution consisting of deionized (DI) water, NH_4_OH and H_2_O_2_ to ensure that no organic material remained attached to the sensor surface. After rinsing with DI water, 100 µL of aqueous 0.01% (*w*/*v*) poly-l-lysine solution (Sigma-Aldrich) was pipetted into the sample reservoir and left for 10 min at room temperature. The reservoir was then rinsed with DI water and dried using N_2_ gas. During the sensor incubation with poly-l-lysine, confluent cells were trypsinized and placed back into suspension with serum containing medium at 37 °C. When the sensor was completely dry, 100 µL of the cell suspension was pipetted into the reservoir and the entire assembly was placed in a 37 °C, 5% CO_2_ incubator for 3 h, allowing the cells to settle and attach onto the sensor surface. After incubation, the remaining solution was carefully aspirated and replaced with warmed experimental buffer solutions. The assembly was then placed in a small incubator capable of maintaining the experimental temperature of 37 °C. The superfusion system was activated with a constant flow of buffer solution until the signal drift reached acceptably low levels (approximately 1 to 3 min). A superfusion sequence consisting of alternating solutions with and without ammonia was initiated to stimulate the cells’ response to changes in intracellular pH (pH_i_). In later experiments, EIPA was used during the unloading phase to inhibit the cells’ response to intracellular acidification through an NHE blockade.

**Figure 3:**
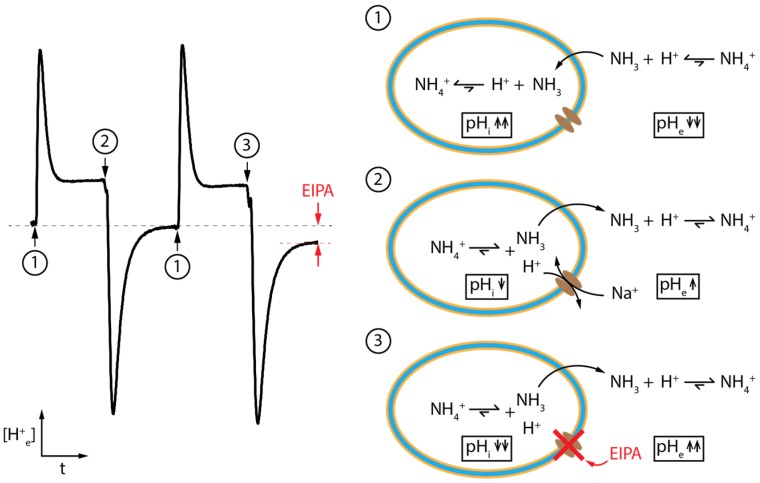
Typical ISFET signals generated during the three individuals intervals. Each interval has a duration of approximately 30 s. The black dotted line indicates the baseline when superfusing with buffered solution. Black arrows indicate the change to the next solution. The red dotted line and red arrows indicate the signal difference caused by the presence of EIPA. The sensor’s signal is proportional to the external proton concentration [H^+^_e_], and, therefore, inversely proportional to the external pH (pH_e_) [7]. Interval 1 is the intracellular alkalinization through exposure to NH_4_^+^. Interval 2 is the intracellular acidification through removal of NH_4_^+^. This activates NHE, causing the release of intracellular H^+^. Interval 3 is similar to interval 2, but with the addition of EIPA, inhibiting the NHE-mediated release of H^+^. The result is a reduction in the [H^+^_e_] and sensor signal as inhibition of NHE generated a larger increase in near-membrane pH_e_ compared to that of interval 2. Note that, like interval 2, interval 3 is preceded by interval 1 in order to restore the cells to their initial condition and generate an acidified intracellular environment.

Figure 3 shows a representative experimental signal generated at the sensor surface during the intervals of ammonia loading and unloading in the absence and presence of EIPA. In the first interval, NH_3_ diffuses rapidly across the plasma membrane, releasing protons at the external surface of the membrane. This proton loss results in a localized decrease in extracellular pH (pH_e_) that is detected by the sensor as illustrated by the upward transient and elevated steady state. Once uncharged NH_3_ diffuses across the bilayer into the cytosol, it rapidly protonates, reducing intracellular free proton concentration and causing an increase in pH_i_. As the membrane bilayer is relatively impermeable to charged protons, only the increase in free protons on the outside of the cell is readily detected by the sensor. Due to the fast reaction kinetics and diffusion rates as well as the limited intracellular volume, the reaction quickly reaches steady-state equilibrium, allowing the next interval to start approximately 30 s after initiating the ammonia loading. In the next interval (2), external ammonia is removed, creating a strong chemical gradient from the intracellular to the extracellular space. This drives cytosolic uncharged NH_3_ out of the cell through diffusion across the plasma membrane where it sequesters free protons outside the cell, transiently raising pH_e_, and, importantly, leaving behind a free proton inside the cell, thus lowering pH_i_. This intracellular acidification in turn facilitates the sodium-coupled transport of NHE, counteracting the intracellular acidification via the exchange of internal H^+^ with external Na^+^. During this phase, the sensor will detect a combination of signals driven primarily at first by the transient diffusion of NH_3_ to the outside where it protonates and briefly increases pH_e_. This is followed by the slower facilitated transport of NHE exchanging extracellular sodium for intracellular proton resulting in an upward voltage shift (decrease in pH_e_) in relation to the initial fast downward voltage shift generated by the transient diffusion of NH_3_. In the third interval, the addition of EIPA specifically inhibits the activity of NHE, allowing analysis of its contribution to the detected signal via comparison with the signal of the second interval. The resulting difference between the second and third interval allows for the detailed examination of contribution to extracellular acidification near the membrane by NHE transport.

## 3. Results and Discussion

### 3.1. Optimization of the Superfusion

**Figure 4:**
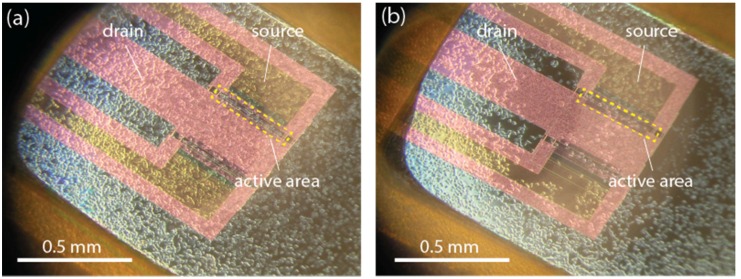
Microscopic photographs of CHO cells attached to the poly-D-lysine coated sensor. The yellow dotted box indicates the active area of the sensor. Images were taken (**a**) before and (**b**) after superfusion. In this case, there was too much shear stress on the cells, causing them to detach during the experiment. Under optimized conditions, there were no significant differences detected between the images taken before and after each experiment.

**Figure 5:**
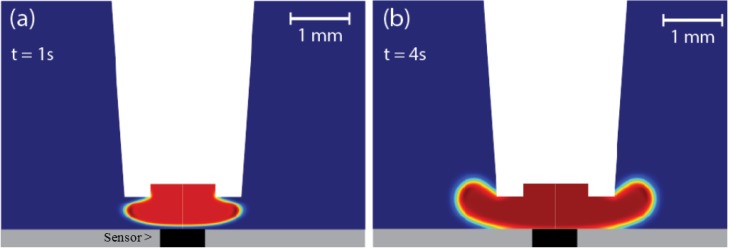
CFD analysis of the flow exiting the pipette (white area) when switching to a new solution. The deep blue area is old solution and the deep red area is new solution. (**a**) shows the distribution of new solution 1 s after switching and (**b**) after 4 s.

In order to obtain the best response from the sensor, we adjusted the distance between the tip of the pipette and the center of the active sensor surface. A shorter distance resulted in an improvement of the solution exchange efficiency and therefore faster signals with higher amplitude. However, the shear stress on the cells increased with decreasing distance and thus increased the risk of cell detachment from the sensor surface (Figure 4). An increase in flow rate had similar effects and it became crucial to optimize the superfusion system using both parameters. In the end, we found a good compromise between flow stability, cell stability and minimal use of solution using a flow rate between 1 and 2 µL/s and a distance of approximately 0.2 mm between tip and sensor surface. To put this into perspective, the largest dimension of the active area of the sensor was approximately 0.3 mm (Figure 4). To verify that the cells had not detached during the experiment, microscopic images of each sample were taken before and after individual experiments. Computational fluid analysis (CFD) using the solvers for the Navier–Stokes equation and Fickian diffusion equation in COMSOL confirmed that quick and efficient solution exchange without turbulent flow is provided at these conditions. In the simulation, the solution leaving the pipette reached the sensor in a little over 1 s, which was deemed acceptable considering the duration of the individual superfusion cycles are a minimum of 30 s (Figure 5).

### 3.2. Ammonia Loading and Unloading

We determined the ideal buffer concentration of the superfusion solutions through assessment of the signals recorded during alternating cycles of ammonia loading and unloading. As expected, lower buffer concentrations resulted in signals with higher amplitude but also reduced their responsiveness due to the slower exchange between the bulk solution and the cell surface. Buffer optimization was dependent not only on signal intensity and speed, but on the fact that lower concentrations of buffer increase the susceptibility to changes in pH when other ions like ammonia, with a relatively high pK_a_ of 9.4, are added and removed.

**Figure 6:**
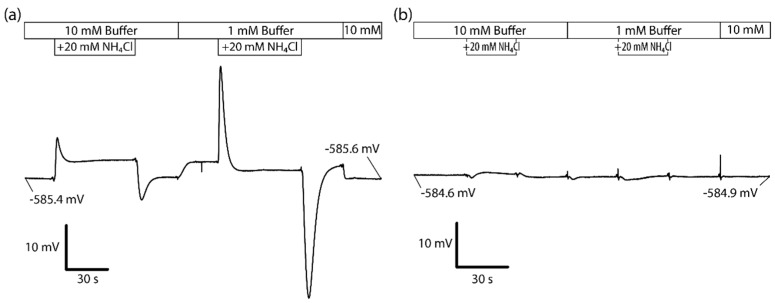
Superfusion sequence utilizing ammonia loading and unloading intervals at 10 mM and 1 mM buffer concentration in the (**a**) presence and (**b**) absence of cells on the sensor.

Figure 6a shows a superfusion sequence using 20 mM NH_4_Cl with 10 mM buffer concentration followed by a 1 mM buffer. The behavior of the signal drift is excellent, amounting to approximately +0.2 mV over a time course of 4 min and 20 s. In combination with a very low noise floor, this allows reliable measurement of signals well below 1 mV. As expected, the transient signals have a much lower amplitude at 10 mM than at 1 mM. This is most likely due to the increased proton exchange efficiency between the cell surface and the bulk solution, giving newly generated or consumed protons at the cell surface less probability for being detected by the sensor. The transient signal during the unloading cycle is stronger than during the loading cycle, which can be explained by the accumulation of intracellular NH_3_. As these are intact cells, intracellular ammonia can continue to rise as there is no cap on the initial rise of the intracellular pH governing the balance between NH_3_ and NH_4_^+^, whereas ammonia and buffer pH are tightly controlled on the outside of the cell. As NH_3_ rapidly diffuses out of the cell, the unloading transient returns to steady-state levels where the rate and signal are primarily controlled by proton flux emanating from the cytosol. Interestingly, the respective steady-state signals differ as well, even though the cell-free control shows no significant difference in the signal between the solutions with and without ammonia (Figure 6b). This effect during the ammonia loading is triggered by a similar mechanism in which cytosolic pH elevation favors protonation of ammonia into NH_4_^+^. This increases the amount of NH_3_ that is available to diffuse into the cell during the steady-state phase as pH is elevated and thus the level of free protons left on the outside of the cell membrane detected by the sensor. A change in the steady-state signal is also observed when the buffer is switched between 1 mM and 10 mM. More protons are detected at 1 mM, which confirms a higher proton concentration near the cell surface than in bulk, as well as an attenuation of the signal at 10 mM due to a higher buffering power of the bulk solution. If there was no difference in the signal, this would implicate that there was either no proton gradient or that such a gradient was not detectable by the sensor. A comparison of the difference in the steady-state signals when switching ammonia reveals that there is different behavior between 1 mM and 10 mM buffer concentration. At 10 mM, there are more protons detected when switching on the ammonia, whereas at 1 mM there is a reduction. We assume that it could be caused by pH regulatory effects of the cells and alterations in transport activity. Loading the cells with uncharged ammonia causes an increase in cytosolic pH due to protonation of the ammonia. This leads to a reduction in activity of NHE, effectively weakening the extracellular proton gradient. This explanation is evident at 1 mM buffer concentration, but at 10 mM, the effect is reversed as the reduction of NHE activity due to elevated cytosolic pH is masked at higher extracellular buffer concentrations. While this could suggest that simple alterations in extracellular buffer may reveal functional output of surface membrane transporters (*i.e.*, decreases in NHE activity) during the ammonia loading phase, it was decided that subsequent analysis of transporter derived proton flux focuses on detecting increases in activity during cellular acidification. To this extent, the unloading phase with 10 mM buffer provided a more physiologically relevant and consistent signal to specifically detect increases in proton flux via NHE.

### 3.3. Repeated Ammonia Cycles and Dependence on Cell Density

**Figure 7:**
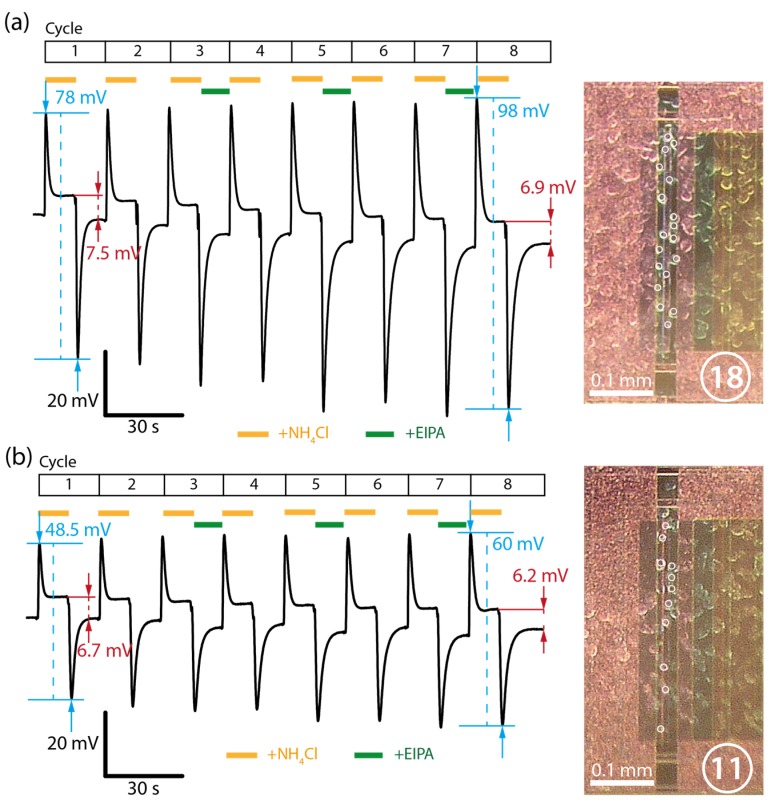
Superfusion sequence of alternating ammonia loading and unloading intervals. Cycles 3, 5 and 7 are unloading intervals in the presence of EIPA. Two representative samples with approximate cell densities of (**a**) 18 cells and (**b**) 11 cells are depicted. White circles mark individual cells.

In order to assess how the signal intensity scales with cell density, two representative experiments were compared with differing cell densities. Figure 7a shows a superfusion sequence conducted on sample with approximately 18 cells attached to the active sensor area. Eight cycles of ammonia loading and unloading were performed with the presence of the NHE inhibitor EIPA during the unloading phase of cycles 3, 5 and 7. Although the influence of EIPA will not be discussed in this section, we found it important to optimize performance and generate conditions directly comparable to the proceeding section. Measuring the peak-to-peak intensity between the loading and unloading phases of a cycle, we found that the signal increased approximately 26% from an initial 78 mV to 98 mV over the course of eight cycles. However, the negative peaks from the unloading phases mainly contributed to this increase, whereas the positive peaks from the loading phases remained virtually constant. As the peak loading signal is generated from controlled exposure of exogenous ammonia, the small decrease in the midline and unloading peak may correspond to a change in the cellular physiological state. Ammonia has been reported to have many long term effects on cell culture and repeated exposure to ammonia may decrease the ability of the cell to maximally extrude cytosolic protons, resulting in a slow decrease in the sensor signal [20]. Consequently, the steady-state difference between the loading and unloading phases decreased by approximately 9% from an initial 7.5 mV to 6.9 mV over the course of eight cycles. If the steady-state signals are indeed dependent on the extracellular proton gradient, a decrease of the signal would suggest a decrease of the cells’ pH regulatory activity.

Figure 7b shows the same experiment performed on a different sample with a lower cell density of approximately 11 cells on the active area of the sensor. Again, we observed an increase of approximately 24% in peak-to-peak intensity, although with lower amplitudes from an initial 48.5 mV to 60 mV after eight cycles. The steady state signals decreased by approximately 8% from an initial 6.7 mV to 6.2 mV after eight cycles. Comparing the cell densities between the two experiments, a proportional relationship with the peak-to-peak intensities is found: an increase in cell density of approximately 75% resulted in an increase in sensor signal of approximately 63%. In the case of the steady-state signals, however, the increase was only 11%. A quantitative relationship between the transient signals and the steady-state signals cannot be established at this stage. However, the general trend that an increased cell density on the sensor results in increased cell-dependent signal levels remains intact.

### 3.4. Amiloride-Sensitivity

**Figure 8:**
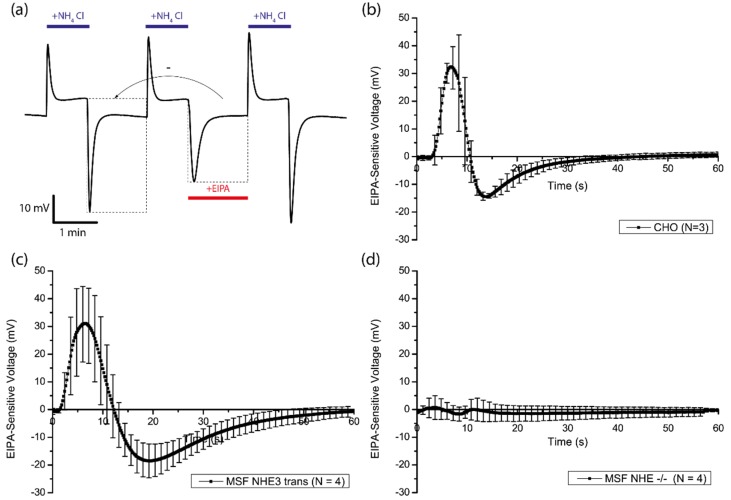
(**a**) superfusion sequence of three cycles of ammonia loading and unloading on CHO cells. The second cycle was performed in the presence of EIPA. Dotted boxes show the data that was used for the subtraction in order to obtain the EIPA-sensitive curves: Averaged curves of (**b**) three CHO samples, (**c**) four NHE3-reconstitued MSF samples and (**d**) four NHE-deficient MSF samples.

To determine the sensor efficacy for evaluation of physiological transporter activity, we focused on the non-electrogenic transport of protons through the sodium-hydrogen exchanger. EIPA is a member of the amiloride class of diuretics that has been classically used for studies involving NHE transport due to its rapid kinetics and exceptional inhibition across most members of the NHE superfamily [18]. The EIPA-sensitive response from Chinese hamster ovary (CHO) cells, NHE-deficient mouse skin fibroblasts (MSF) and NHE-deficient mouse skin fibroblasts reconstituted with NHE3 was evaluated. In order to obtain the EIPA-sensitive response, the dataset of the ammonia unloading phase was subtracted from the dataset of the ammonia unloading phase in the presence of EIPA (Figure 8a). Figure 8b shows the average EIPA-response of three CHO samples. The signal is composed of two main transients, peaking at +33 mV after 7 s with a rapid drop thereafter, bottoming out at −15 mV after 14 s and finally transitioning into an asymptotic recovery phase. This suggests the presence of two mechanisms: (a) an initial fast transient characterized by an EIPA-dependent decrease in the transient alkalinization of the extracellular space; (b) a slower response in the opposite direction as the cytosol becomes acidic indicating an increase in the near-surface proton flux emanating from the cell. As the second slower response is characterized by a low cytosolic pH gradient, it is clear that this reduced sensor signal is attributed to the inhibition of the canonical mode of transport of NHE, namely the exchange of Na^+^ with H^+^ as both ions are strongly favored for transport along the chemical gradients. What is less clear is the interpretation of the initial fast transient demonstrating an EIPA-sensitive decrease in extracellular basification. During the first transient, the sensor indicates a strong basification on the extracellular space, which is predominantly non-EIPA-sensitive. This is due to the rapid diffusion of intracellular NH_3_ across the bilayer during washout. Once in the extracellular space, NH_3_ is rapidly protonated to NH_4_^+^, generating the basification indicated by the sensor. EIPA should not directly effect the diffusion of ammonia across the bilayer and there should be no signal difference during this phase; however, it is clear that this response is attenuated by the presence of EIPA. One possibility of how EIPA could mitigate this response is through the non-canonical transport of NH_4_^+^ in exchange for sodium via NHE. It has been demonstrated previously that NH_4_^+^ can be transported by directly competing with cytosolic proton binding during NHE exchange [11,21,22,23]. Moreover, extracellular NH_4_^+^ is unlikely to be deprotonated and detected by the sensor due to its low dissociation constant of around 1% at near physiological pH [23]. This suggests that under EIPA-free conditions there is a partial contribution of NHE toward the basification of the extracellular space as NHE favors the transport of NH_4_^+^. This transient mode would only be possible during the initial moments of washout when cytosolic pH is high and passive diffusion of NH_3_ has not yet led to a rapid drop in both pH and free ammonia levels. The NHE dependent transport of charged ammonia out of the cell slows down the acidification process and allows more uncharged NH_3_ to diffuse out of the cell, become protonated and generate an increased drop in sensor signal. Blockage of NHE through EIPA at this time appears to cause extracellular acidification, converse to its normal mode of operation, but is likely due to NHE transport of NH_4_^+^ being blocked, causing alterations in NH_3_ diffusion rates. After the ammonia has been driven out of the cells, free protons remain in the cytosol, activating the main mode of NHE (second transient) and the sensor predominantly detects EIPA-dependent extracellular acidification from proton transport across NHE.

The EIPA response in MSF reconstituted with NHE3 showed a similar pattern to the CHO cells, with a comparable peak value for the first transient, but a more pronounced recovery phase thereafter (Figure 8c). As the recovery phase is indicative of proton transport through NHE, differences in this phase are likely due to factors that affect the expression level and function of NHE in a cell-type specific manner as well as physiological characteristics of individual cell types such as surface area to volume ratios and intracellular buffering capabilities. In NHE-deficient MSF, no significant EIPA-response was recorded (Figure 8d), as would be expected from cells with no means for H^+^/Na^+^ exchange.

## 4. Conclusions

In this article, we described a system for measuring the pH response of mammalian cells in close proximity to the plasma membrane using an ISFET sensor and a continuous superfusion system. We obtained fast, stable and strong pH signals from CHO cells and MSF using BTP as a buffer agent at 1 mM and 10 mM. Short intervals of ammonia loading and unloading proved to be a viable method for stimulating cellular pH response via intracellular alkalinization through NH_3_ loading followed by rapid intracellular acidification during washout. Optical observations before and after experimentation revealed no obvious visual markers for cell membrane stress including membrane shedding, blebs or the formation of large intracellular puncta even after many repetitions of these cycles. However, a gradual shift of the transient signal occurred, possibly as a result of ammonia generating a successive and altered cellular response to pH stress [20]. The amplitude of the signal was also found to be dependent on the cell density at the sensor surface. Furthermore, cell-dependent steady-state signals were detected, suggesting that the ISFET sensor was not only capable of recording transient signals but also the extracellular proton-gradient. Finally, a series of experiments using EIPA as a potent blocking agent for NHE was conducted to isolate the NHE-dependent transport during the unloading of ammonia. We found that CHO cells as well as NHE-deficient MSF reconstituted with NHE3 responded positively to EIPA. NHE-deficient MSF exhibited virtually no response. To our surprise, the positive EIPA-responses consisted of multiple transients whose mechanisms remain to be fully elucidated but appear related to reports indicating the capability of direct ammonium transport through NHE. Nevertheless, this work may lay the foundation for further investigation regarding the evaluating of agents acting on pH-regulatory pathways. The next important steps would include experimentation to obtain dose-response curves and investigation of other proton-sensitive membrane proteins such as ATP-dependent proton-pumps. This would open the possibility for many applications furthering our basic knowledge of the fast transport kinetics of many difficult to evaluate transporters and channels. Due to the method’s relative low cost combined with scalability, speed and reliability, these techniques could generate novel applications in drug discovery, screening and diagnostics for a myriad of clinically relevant disciplines.
